# Crosstalk between the mesothelium and lymphomatous cells: insight into the mechanisms involved in the progression of body cavity lymphomas

**DOI:** 10.1002/cam4.159

**Published:** 2013-11-19

**Authors:** Laura Lignitto, Adriana Mattiolo, Elena Negri, Luca Persano, Lisa Gianesello, Luigi Chieco-Bianchi, Maria Luisa Calabrò

**Affiliations:** 1 Immunology and Molecular Oncology, Veneto Institute of Oncology, IOV—IRCCSPadova, Italy; 2 Department of Women's and Children's Health, University of PadovaPadova, Italy; 3 Department of Surgery, Oncology and Gastroenterology, University of PadovaPadova, Italy

**Keywords:** Body cavity, EMT, lymphoma, mesothelial cells, microenvironment

## Abstract

The peculiar localization of body cavity lymphomas implies a specific contribution of the intracavitary microenvironment to the pathogenesis of these tumors. In this study, primary effusion lymphoma (PEL) was used as a model of body cavity lymphoma to investigate the role of mesothelial cells, which line the serous cavities, in lymphoma progression. The crosstalk between mesothelial and lymphomatous cells was studied in cocultures of primary human mesothelial cells (HMC) with PEL cells and a xenograft mouse model of peritoneal PEL. PEL cells were found to induce type 2 epithelial–mesenchymal transition (EMT) in HMC, which converted into a myofibroblastic phenotype characterized by loss of epithelial markers (pan cytokeratin and E-cadherin), expression of EMT-associated transcriptional repressors (Snail1, Slug, Zeb1, Sip1), and acquisition of α-smooth muscle actin (α-SMA), a mesenchymal protein. A progressive thickening of serosal membranes was observed in vivo, accompanied by loss of cytokeratin staining and appearance of α-SMA-expressing cells, confirming that fibrosis occurred during intracavitary PEL development. On the other hand, HMC were found to modulate PEL cell turnover in vitro, increasing their resistance to apoptosis and proliferation. This supportive activity on PEL cells was retained after transdifferentiation, and was impaired by interferon-α_2_b treatment. On the whole, our results indicate that PEL cells induce type 2 EMT in HMC, which support PEL cell growth and survival, providing a milieu favorable to lymphoma progression. Our findings provide new clues into the mechanisms involved in lymphoma progression and may indicate new targets for effective treatment of malignant effusions growing in body cavities.

## Introduction

Body cavity lymphomas are a heterogeneous group of non-Hodgkin's lymphomas that primarily grow as recurrent effusions in large serous body cavities generally in the absence of a solid tissue mass. These lymphomas include primary effusion lymphoma (PEL), associated with human herpesvirus 8 (HHV8) infection 1–3, pyothorax-associated lymphoma, linked to Epstein–Barr virus (EBV) infection, and characterized by pleural effusion and/or a mass with infiltration of the pleura 4, and lymphomas developing in patients with liver cirrhosis associated with hepatitis B virus (HBV) or hepatitis C virus (HCV) infection 5. Cases of body cavity lymphoma not associated with viral infections have also been described 6,7. Why these lymphomatous entities preferentially develop in body cavities is not fully understood. It has been proposed that a condition of persistent inflammation may promote the activation of the mesothelium lining the body cavities. Activated mesothelial cells may in turn generate a milieu rich in chemokines, growth factors, and adhesion molecules that may mediate the homing and support the proliferation of uninfected or virus-infected lymphocytes or lymphomatous precursors within body cavities. However, while the influence of bone marrow milieu in malignant hematopoietic disorders is well studied 8, the role of serous cavities, and specifically the mesothelium, in initiation and progression of body cavity lymphomas has not yet been elucidated in depth.

Mesothelial cells are specialized epithelial-like cells that control intracavitary homeostasis by regulating inflammatory processes, tissue repair, and fluid, and molecule transport across the serous membranes 9,10. Human mesothelial cells (HMC) also provide a frictionless interface for intracoelomic movement through secretion of several extracellular matrix components. HMC are per se cells endowed with high plasticity, and they can modulate their morphology and secretory repertoire in response to different conditions. Epithelial–mesenchymal transition (EMT) of HMC occurs in patients undergoing peritoneal dialysis and this process leads to peritoneal fibrosis with serosal thickening and functional deterioration 11–14. While serosal thickening was evidenced with computed tomography scans in patients affected by primary body cavity lymphomas as well as peritoneal lymphomatosis 15–17, the association with EMT has not yet been reported.

EMT is a complex, reversible, and stepwise biological process involving a functional transition of static and polarized epithelial cells into mobile mesenchymal cells 18,19. EMT is classified into three different types. Type 1 EMT occurs during implantation, embryogenesis, and organ development. Inflammation drives type 2 EMT, which is mainly involved in wound healing and tissue regeneration; persistent inflammatory stimuli may lead to tissue fibrosis and eventually to organ destruction. Activation of type 3 EMT mediates the invasive and metastatic behavior of epithelial and endothelial cancers.

PEL is a highly aggressive B-cell lymphoma predominantly localized within body cavities (pleural, peritoneal, and pericardial) that develops more frequently in human immunodeficiency virus–infected patients 1–3. PEL has also been linked to other immunosuppressive conditions, such as solid organ transplantation, cancer, old age, and HBV-or HCV-associated liver cirrhosis. PEL was first recognized as a distinct clinicopathological entity in the World Health Organization classification of neoplastic diseases of the hematopoietic and lymphoid tissues in 2001 20,21. Histologically, PEL is characterized by atypical lymphoid cells of B-cell origin with large nuclei and prominent nucleoli. Demonstration of HHV8 in the tumor clone is required for PEL diagnosis. A subset of PEL patients may simultaneously or secondarily develop solid tumors in structures adjacent to body cavities 22. HHV8 was also detected in lymphoma cases presenting as tissue masses (“extracavitary” PEL) and exhibiting morphology, immunophenotype, and gene expression profiles similar to PEL 2,23. Moreover, HHV8-positive polyclonal effusions were also shown in patients affected by other HHV8-associated pathological conditions 24, suggesting that the spectrum of PEL may be wider than that defined initially. Once established, PEL is irreversible, rapidly progressive, and consistently lethal. The presence of several comorbidities in PEL patients makes the therapeutic options very limited, as conventional antineoplastic chemotherapy is often not feasible in the majority of immunocompromised or elderly patients. Previous studies showed that intracavitary targeting of the murine microenvironment with a lentiviral vector expressing murine interferon-α_1_ (mIFN-α_1_) exerted a significant antineoplastic activity in a xenograft mouse model of PEL 25. These findings highlighted the relevance of the local milieu in PEL growth.

In this study, we investigated the role of mesothelial cells in lymphoma progression using PEL as a model of body cavity lymphoma. Cocultures of primary HMC with PEL cells were used to reproduce in vitro some of the heterotypic interactions existing in body cavities and to analyze the crosstalk between lymphomatous and mesothelial cells. Furthermore, a xenograft mouse model was used to study the modifications of serosal membranes during in vivo PEL progression. Our data show that PEL cells induce EMT of HMC and thickening of serosal membranes during intracavitary PEL growth. In turn, HMC and their transdifferentiated counterparts enhance PEL cell survival and growth, providing an environment favorable to PEL progression. IFN-α_2_b treatment of cocultures appears to counteract the supportive behavior of HMC. These findings provide new insights into the mechanisms governing body cavity lymphoma progression.

## Materials and Methods

### Cell lines

The EBV−/HHV8+ CRO-AP/3 and EBV+/HHV8+ CRO-AP/2 PEL-derived cell lines were grown in RPMI 1640 (Sigma-Aldrich, Munich, Germany) supplemented with 20% fetal calf serum (FCS, Gibco, Life Technologies, Foster City, CA) and 2 mmol/L l-glutamine (Gibco, Life Technologies) (complete medium). The EBV−/HHV8+ BCBL-1 PEL-derived cell line was grown in RPMI 1640 supplemented with 10% FCS, 2 mmol/L l-glutamine, 50 μg/mL gentamycin (Sigma-Aldrich), 100 U/mL penicillin, and 100 μg/mL streptomycin (Life Technologies). All cell lines were authenticated, and mycoplasma free, as resulting from periodical polymerase chain reaction (PCR) check.

### Isolation of peritoneal mesothelial cells

Primary HMC were obtained from visceral effusions from patients affected by nonviral-related liver cirrhosis and from human peritoneal fragments from patients undergoing abdominal surgery for non-neoplastic diseases. Approval of the Institutional Ethical Committee was obtained for the use of these biological materials and informed consent was obtained from all patients.

Visceral effusions from patients affected by nonviral-related liver cirrhosis were twofold diluted with DMEM-F12 (Sigma-Aldrich), centrifuged for 15 min at 200*g*, washed twice with normal medium, and suspended to a final concentration of 1.2–1.5 × 10^5^ cells/mL in DMEM-F12 supplemented with 5% FCS, 2 mmol/L l-glutamine, 50 μg/mL gentamycin, 100 U/mL penicillin, 100 μg/mL streptomycin, and 0.25 μg/mL amphotericin B (Life Technologies). Cell suspensions were transferred to 75 cm^2^ tissue culture flasks for 30 min in a 37°C, 5% CO_2_ humidified incubator for monocyte depletion, and then transferred to poly-l-lysine-coated (molecular weight 70,000–150,000, 0.01%) (Sigma-Aldrich) 75 or 150 cm^2^ flasks. Primary HMC from peritoneal fragments were obtained as previously reported 25. At confluence, HMC were used to set up the cocultures with PEL-derived cell lines. Phenotypic characterization of HMC was performed by flow cytometry analyses (Fig. S1) and indirect immunofluorescence assays (IFA).

To evaluate the expression of markers modulated during EMT by qualitative and quantitative reverse transcription PCR (RT-PCR), subconfluent first-passage HMC were cocultured with PEL-derived cells lines for 6 days, then PEL cells were removed. HMC were washed twice with normal medium, detached by trypsin/EDTA treatment, washed twice with cold phosphate-buffered saline (PBS, Oxoid, Basingstoke, UK) and processed for total RNA extraction. As a positive control, HMC were induced to undergo EMT by treatment with recombinant transforming growth factor-β1 (TGF-β1, 1 ng/mL), and interleukin-1β (IL-1β, 2 ng/mL) (R&D Systems, Minneapolis, MN) for 4 days. HMC undergoing EMT (EMT–HMC) were also used in coculture systems to assess PEL cell turnover. To analyze pan cytokeratin (pCK) and α-smooth muscle actin (α-SMA) expression in HMC by IFA, cocultures with CRO-AP/3 cells were prolonged for up to 12 days. Mesothelial cells cultured in the different experimental conditions were visualized using an optical microscope (Leica DMIL LED, Leica Microsystems, Wetzlar, Germany) with a 4×/0.10 NA objective, and images were acquired with a digital color camera (EC3, Leica Microsystems) using the Leica Acquisition Suite (LAS) EZ software (version 2.0.0, Leica Microsystems). Images were visualized and adjusted using CorelDRAW Graphics Suite X3 (Corel Corporation, Ottawa, Ontario, Canada). CRO-AP/3 cells were also cocultured with HMC in transwell chambers to assess whether EMT induction was mediated by soluble factors. SB-431542 (Sigma-Aldrich) (5 μmol/L and 10 μmol/L) was added to cocultures to selectively inhibit the TGF-β1 receptor 26.

### Molecular analyses

Total RNA was extracted using RNA-Bee reagent (Tel-Test Inc., Friendswood, TX) according to the manufacturer's protocol. First-strand cDNA synthesis was performed with 1 μg of total RNA using SuperScript II Reverse Transcriptase (Life Technologies), as previously reported 27. Expression of E-cadherin and Snail1 was evaluated by qualitative and quantitative RT-PCR analyses. Primer sets used in qualitative RT-PCR are reported in Table S1. Human β-actin expression was analyzed in parallel as control. After an initial 8-min DNA denaturation and Taq polymerase activation, 40 cycles were run at 94°C for 1 min, 56°C (for E-cadherin-specific primers) or 58°C (for Snail1 specific primers) for 1 min, and 72°C for 1 min; these were followed by a 7-min extension step at 72°C. Each PCR sample was analyzed by electrophoresis on a 2% agarose gel and visualized by SYBR Safe DNA gel staining (Life Technologies). Quantitative real-time RT-PCR for human porphobilinogen deaminase (PBGD, housekeeping gene), pCK, E-cadherin, Snail1, Slug, Zeb1, Sip1, α-SMA, TGF-β, and tumor necrosis factor–related apoptosis-inducing ligand (TRAIL) expression was performed with intron-spanning primer pairs (Table S1). Reactions were carried out using Platinum SYBR Green qPCR SuperMix-UDG (Life Technologies) in a Sequence Detection System SDS 7900HT (Applied Biosystems, Life Technologies). Melting curves were analyzed for each reaction to ensure the specificity of the amplicons. Data elaboration was performed as relative quantification analysis using the ΔΔCt method.

### Mice

Six-week-old female severe combined immunodeficiency (SCID)/CB17 mice were purchased from Charles River Breeding Laboratories (Calco, Italy), housed under specific pathogen-free conditions in the BL2 containment laboratory in our animal facility, and allowed to acclimate to local conditions for 1 week. Procedures involving animals and their care were in conformity with the institutional guidelines that comply with the Italian Animal Welfare Law (D.L. n. 116/1992; and subsequent documents). The project was approved by the local ethical committee of the University of Padova (Comitato Etico di Ateneo per la Sperimentazione Animale, CEASA) and communicated to the relevant Italian authority (Italian Ministry of Health, VI Office).

### Assessment of the mesothelium response in SCID/PEL mice

To characterize the mesothelium response in vivo, SCID mice were intraperitoneally injected with 50 × 10^6^ of logarithmically growing CRO-AP/3 cells. At this dose, a liquid-phase tumor usually engrafts 6–10 days from PEL cell inoculum, with the development of a consistent ascites within the same time interval 25. Mice were sacrificed at 8 and 12 days after cell inoculation to evaluate mesothelium response. Fragments of visceral mesothelium and diaphragms were collected, fixed in 4% formalin, paraffin-embedded, and cut in 5-μm-thick sections. Hematoxylin–eosin staining was used for histological examinations. Sections were deparaffined and rehydrated, and antigen retrieval was performed by incubation with citrate buffer 0.01 mol/L (pH 6.0) at 95°C for 20 min. After saturation with 2% normal goat serum, slides were incubated with primary antibodies to pCK and α-SMA, then washed and incubated with appropriate Alexa dye–conjugated secondary monoclonal antibodies (mAbs). Stained sections were visualized and images were acquired as stated above.

### Statistical analyses

Student *t* test was used to estimate statistical significance of differences between two groups. One-way analysis of variance (ANOVA) was applied to assess the statistical significance of differences between groups. *P* values <0.05 were considered significant. Details about other techniques and reagents are reported in Data S1.

## Results

### Coculture with PEL cells induces a myofibroblastic morphology in mesothelial cells

**Figure 1:**
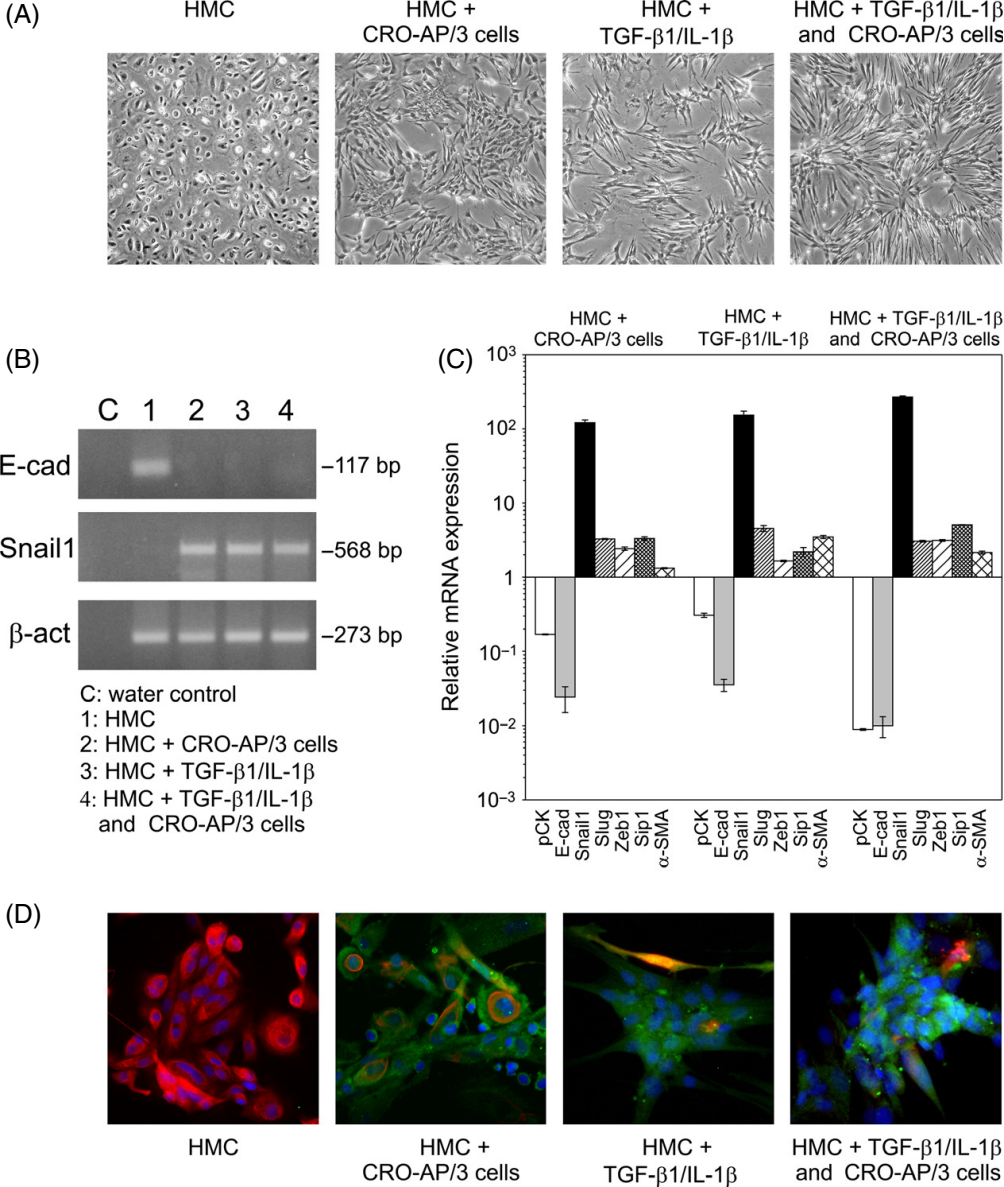
CRO-AP/3 cells induce EMT in mesothelial cells. (A) Phase-contrast images of representative HMC in standard culture conditions and converted into a myofibroblastic morphology after coculture with CRO-AP/3 cells for 6 days, or after 4 days of TGF-β1/IL-1β treatment (positive control for EMT induction), or after 2 days of TGF-β1/IL-1β treatment followed by 2 days of coculture (combined condition), respectively. Original magnifications 4×. (B) Analysis of E-cadherin (E-cad) and Snail1 transcripts in HMC cultured in the four conditions reported above was performed by qualitative RT-PCR. Expression of β-actin (β-act) is shown below as control. (C) Relative expression of transcripts encoding E-cad, pCK, Snail1, Slug, Zeb1, Sip1, and α-SMA was determined by real-time RT-PCR in HMC in the four culture conditions. PBGD was used as housekeeping gene to normalize the variability in template loading. Expression levels of the analyzed transcripts are reported relative to expression levels measured in HMC cultured in standard conditions. Data are reported as mean ± SD. (D) HMC, placed in the four culture conditions for longer time periods, were analyzed for the expression of pCK (red) and α-SMA (green) by IFA, with 4',6-diamino-2-phenylindole (DAPI) (blue) counterstain. HMC cultured in standard conditions show a uniform layer of pCK-positive cells. HMC in the other three conditions show reduced pCK expression and the presence of α-SMA-positive cells. α-SMA-positive cells were detected only very rarely in HMC cultured in standard conditions. Original magnifications 20×. EMT, epithelial–mesenchymal transition; HMC, human mesothelial cells; TGF-β1, transforming growth factor-β1; IL-1β, interleukin-1β; RT-PCR, reverse transcription polymerase chain reaction; pCK, pan cytokeratin; α-SMA, α-smooth muscle actin; PBGD, porphobilinogen deaminase; IFA, immunofluorescence assay.

As mesothelium may go through different architectural alterations in response to numerous stimuli, we first assessed whether coculture with the CRO-AP/3 PEL-derived cell line could affect HMC morphology. To this end, subconfluent primary cultures of HMC were cocultured with CRO-AP/3 cells for up to 12 days. HMC were found to spindle and pile up, reaching a pattern of multilayered crisscross growth (Fig. 1A). The transition to a myofibroblastic morphology occurred in 5–6 days. Parallel control cultures of HMC, plated at the same concentration and maintained in complete medium, reached a typical flat, cobblestone morphology, without signs of three-dimensional outgrowth. As a positive control, HMC were treated with TGF-β1 and IL-1β, two potent inducers of EMT in HMC 13. Although TGF-β1 alone induces EMT, IL-1β was shown to potentiate EMT by stimulation of autocrine secretion of TGF-β1 28. TGF-β1/IL-1β-treated HMC showed a transition to a myofibroblastic morphology in 4 days similar to that achieved during coculture. HMC were also TGF-β1/IL-1β-pretreated for 48 h, washed, and then cocultured with CRO-AP/3 cells. This combined condition led to a much quicker transition to a myofibroblastic morphology, being reached after 48 h of coculture (Fig. 1A). This finding indicates that TGF-β1/IL-1β treatment cooperates with the coculture, suggesting that a preexisting activation status of the mesothelial lining may predispose to a more rapid transdifferentiation. Coculture with other PEL cell lines (BCBL-1, CRO-AP/2) reproduced the transition to a myofibroblastic morphology (Fig. S2A), indicating that all PEL cell lines may induce transdifferentiation in HMC.

### PEL cells promote EMT in mesothelial cells

We next evaluated whether these morphological changes were consistent with EMT. A central modulator of EMT is Snail1, a potent transcriptional repressor of E-cadherin, the predominant cell–cell adhesion molecule present on nearly all epithelial cell types 29. During all EMT processes, Snail1 expression is highly induced and, consequently, E-cadherin is downregulated. We first assessed E-cadherin and Snail expression by qualitative RT-PCR analyses in HMC cocultured with CRO-AP/3 cells. RT-PCR analyses showed E-cadherin expression in HMC under normal culture conditions, whereas HMC cocultured with CRO-AP/3 showed its downregulation together with the induction of Snail1, an expression profile similar to that obtained in the positive control and in the combined condition (Fig. 1B).

The expression profile of markers modulated during EMT was analyzed by quantitative RT-PCR. The coculture with CRO-AP/3 cells repressed expression of epithelial markers (pCK and E-cadherin), increased expression of EMT-related transcription repressors (Snail1, Slug, Zeb1, and Sip1), and upregulated the mesenchymal marker α-SMA in HMC (Fig. 1C). This expression profile was similar to that achieved by TGF-β1/IL-1β stimulation. An additive effect was measured in the majority of analyzed markers in the combined condition, suggesting that EMT might be activated with similar mechanisms. A comparable modulation of pCK and Snail1 expression was measured in HMC cocultured with BCBL-1 and CRO-AP/2 cells (Fig. S2B).

IFA confirmed de novo synthesis of α-SMA after myofibroblastic conversion in HMC cocultured with CRO-AP/3 cells for 12 days, or stimulated by TGF-β1/IL-1β for 8 days, and in the combined condition for a total of 8 days (Fig. 1D). In parallel, coculture with CRO-AP/3 cells, TGF-β1/IL-1β treatment, and the combined condition downregulated the expression of pCK, expressed in normal HMC (Fig. 1D). Collectively, these results indicate that PEL cells can induce EMT in mesothelial cells.

### TGF-β1 secreted by PEL cells induces EMT in mesothelial cells

**Figure 2:**
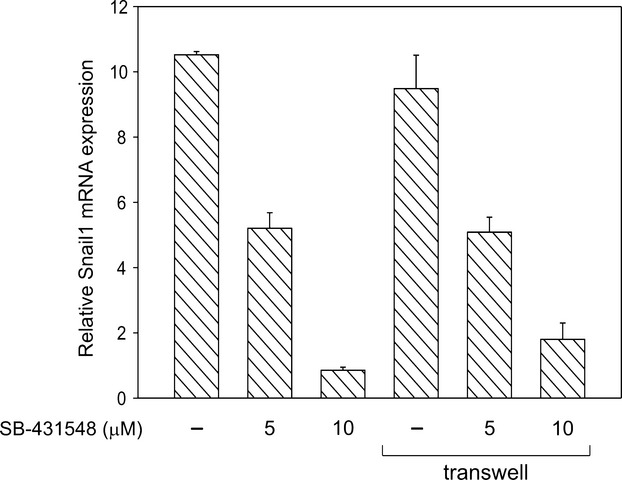
Relative expression of Snail1 transcripts was measured by real-time RT-PCR in HMC cocultured for 24 h with CRO-AP/3 cells, either in direct contact or placed in transwell chambers. PBGD was used as housekeeping gene and data are reported as mean ± SD. Snail1 expression levels are relative to those of HMC cultured in standard conditions. Snail1 induction was similar in both culture conditions, indicating that EMT is triggered by a soluble factor. Snail1 expression was reduced by SB-431548 treatment in a dose-dependent manner, suggesting that increase in Snail1 expression is mainly mediated by TGF-β1 released by CRO-AP/3 cells. RT-PCR, reverse transcription polymerase chain reaction; HMC, human mesothelial cells; PBGD, porphobilinogen deaminase; EMT, epithelial–mesenchymal transition; TGF-β1, transforming growth factor-β1.

PEL-derived cell lines were previously shown to secrete TGF-β1, a potent fibrotic stimulus for HMC as well as a master regulator of EMT 30,31. We confirmed that our PEL cell lines expressed and secreted this factor at high levels (Fig. S3), and analyzed whether EMT in HMC was mediated by a soluble factor. Cocultures were set up in parallel with CRO-AP/3 cells added directly to the culture or placed in transwell chambers, and increase in Snail1 transcription in HMC was used as an early marker of EMT induction. As shown in Figure 2, levels of Snail1 transcripts were similar in both coculture conditions after 24 h, indicating that EMT was induced by factor(s) secreted by PEL cells in culture supernatants. Parallel cocultures with paraformaldehyde-fixed CRO-AP/3 cells, and therefore not producing TGF-β1, did not induce Snail1 expression (data not shown). Prolonged coculture in transwell inserts also induced a myofibroblastic morphology and an increase in other EMT-associated transcription factors in HMC (data not shown). Coculture in the presence of SB-431542, a specific inhibitor of TGF-β signaling 26, showed a dose-dependent suppression of Snail1 expression (Fig. 2), suggesting that EMT in HMC is mainly a TGF-β1-mediated process.

### Analysis of the mesothelium response in vivo

**Figure 3:**
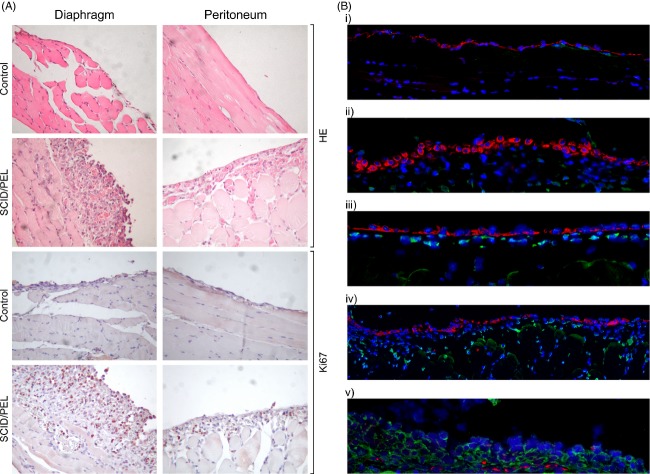
Analysis of EMT in the mesothelium in a SCID/PEL preclinical model. (A) Histological and immunohistochemical analyses of the serosal membranes during in vivo ascites growth. Hematoxylin–eosin (HE) staining was used for histological examinations. Representative photomicrographs show a uniform monolayer of mesothelial cells in serosal membranes from control mice (top row). SCID/PEL mice (day 12) show discontinuity in the mesothelial lining and thickening of the submesothelial region (second row), with new enlarged capillaries containing erythrocytes. Ki67-positive cells, indicative of a hyperproliferative process, are evident in the mesothelial and submesothelial regions in tissue sections from SCID/PEL mice (bottom row). Sections from control mice (third row) do not show Ki67-positive cells. Original magnifications 20×. (B) Detection of pCK and α-SMA by IFA. Representative images of parietal peritoneal fragments from control mice (i and ii) show a continuous and flat monolayer of pCK-positive (red) mesothelial cells at the surface of the peritoneum, and specific staining for α-SMA is not evident. Tissue sections from SCID/PEL mice (day 8) show a progressive increase in α-SMA-positive (green) cells in the submesothelial region, with a more and more irregular layer of pCK-positive cells at the surface of the peritoneum (iii and iv). SCID/PEL mice with maximum abdominal distension (day 12) show an increased number of α-SMA-positive cells, almost complete loss of pCK-expressing cells at the surface, and the appearance of some pCK-positive cells in the interstitial area (v). Original magnifications 20×. EMT, epithelial–mesenchymal transition; SCID/PEL, severe combined immunodeficiency/primary effusion lymphoma; pCK, pan cytokeratin; α-SMA, α-smooth muscle actin; IFA, immunofluorescence assay.

To test whether EMT may also occur in vivo, we used a xenograft SCID mouse model that mimics the liquid phase and aggressive course of PEL 25. Mice were sacrificed 8 and 12 days after PEL cell inoculation, and peritoneal and diaphragm fragments were analyzed to monitor the status of serosal membranes during intracavitary PEL growth. Histological examination revealed a hyperplastic process involving the serosal membranes (Fig. 3A). Granulation tissue with an infiltrate containing mononuclear cells and macrophages, and new dilated capillaries containing erythrocytes were evidenced. Thickening of serosal membranes accompanied by inflammation and neoangiogenesis was suggestive of fibrosis. A proliferative process was confirmed by Ki67 staining (Fig. 3A).

As peritoneal thickening was found to be induced by EMT in patients undergoing peritoneal dialysis 11–14, we analyzed the pattern of pCK and α-SMA expression in the parietal peritoneum of SCID/PEL mice by IFA (Fig. 3B). In a control mouse, the peritoneum showed a flat, continuous monolayer of mesothelial cells, positive for pCK. During ascites progression, the mesothelial lining showed discontinuous tracts, with the appearance of α-SMA-positive cells in the submesothelial region. Mice sacrificed at 12 days showed the loss of cytokeratin signal at the surface of the peritoneum, with an increased number of α-SMA-positive cells as well as some pCK-positive cells in the submesothelial region. These data indicate that EMT of mesothelial cells leading to fibrosis occurs during ascites progression in a preclinical model of PEL.

### HMC and EMT-HMC modify PEL cell turnover

**Figure 4:**
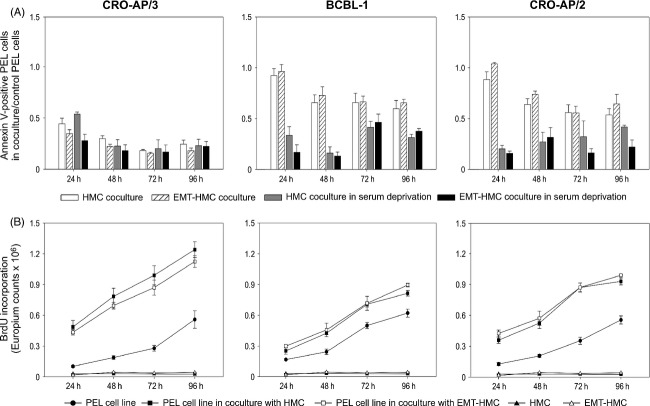
HMC and EMT-HMC modulate PEL cell turnover. (A) Total spontaneous apoptosis was quantified in PEL cells cocultured with HMC (□) and EMT-HMC (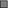). Apoptosis was also induced by serum deprivation and analyzed in PEL cells cocultured with HMC (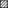) and EMT-HMC (▀). Apoptosis is reported as a ratio between the mean percentage of annexin V–positive PEL cells in three cocultures and the mean percentage of total apoptosis of control cells. Therefore, values less than 1 show a protective effect against apoptosis. SD of the ratio is calculated according to the theory of error propagation, as previously reported 25. A protective activity was present across all cell lines, except at 24 h in the noninduced BCBL-1 and CRO-AP/2 conditions, where no protection was observed (Student *t* test, *P *<* *0.05; CRO-AP/3 in coculture vs. control cell line, all analyzed time points; BCBL-1 or CRO-AP/2 in coculture vs. control cell line, all analyzed time points except 24 h for spontaneous apoptosis only). (B) BrdU incorporation was measured in PEL cells in coculture with HMC (▀) or EMT-HMC (□) and in control cells (•). Data are expressed as Europium counts × 10^6^ and are reported as the mean ± SEM obtained in two independent experiments set up in triplicates. BrdU uptake of HMC (▴) and EMT-HMC (△) seeded at the same concentration as coculture is also shown. HMC, human mesothelial cells; EMT, epithelial–mesenchymal transition; PEL, primary effusion lymphoma; BrdU, 5-bromo-2′-deoxyuridine.

As body cavities are the preferential site of PEL development, they may favor PEL cell survival. We therefore investigated whether mesothelial cells may modify PEL cell turnover. Spontaneous and induced apoptosis was analyzed for 4 days by flow cytometry after staining with annexin V. Apoptosis in PEL cell lines was induced by serum deprivation. All PEL cell lines showed a statistically significant decrease in spontaneous and induced apoptosis after coculture with HMC and EMT-HMC compared with the corresponding control cell line. BCBL-1 and CRO-AP/2 cells showed a significant reduction in spontaneous apoptosis only after 48 h of coculture (Fig. 4A).

Proliferation of PEL cells, analyzed by 5-bromo-2′-deoxyuridine (BrdU) incorporation, was also significantly increased in coculture with HMC and EMT-HMC (ANOVA, *P *≤* *0.01, PEL cells in coculture vs. control PEL cells) (Fig. 4B). BrdU incorporation of HMC and EMT-HMC, seeded at the same concentration as coculture, was minimal. These findings suggest that HMC may support PEL cell growth and survival, thus providing a milieu favorable to PEL progression. Interestingly, this supportive activity was retained by transdifferentiated HMC.

### IFN-α_2_b counteracts the prosurvival activity exerted by HMC and EMT-HMC on PEL cells

**Figure 5:**
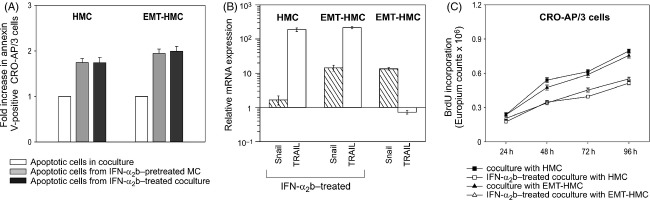
IFN-α_2_b treatment impairs the supportive activity of HMC or EMT-HMC on PEL cells. (A) Apoptosis was analyzed in CRO-AP/3 cells from cocultures with IFN-α_2_b-pretreated mesothelial cells (MC) 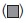 or from IFN-α_2_b-treated cocultures (▀). Data are reported as ratio between the mean of the percentage of total annexin V–positive PEL cells in three different cocultures with IFN-α_2_b treatment and the mean of total apoptotic cells in control untreated cocultures (□), and the SD of the ratio are calculated as stated in the legend to Figure 4A. (B) Relative expression of Snail1 and TRAIL transcripts was measured in IFN-α_2_b-treated HMC and EMT-HMC. Snail1 expression is shown as an EMT-associated marker. PBGD was used as housekeeping gene and expression levels are relative to levels measured in untreated HMC. Data were obtained in six different experiments and are reported as mean ± SEM. (C) CRO-AP/3 cell proliferation was quantified in cocultures with HMC (▀) or with EMT-HMC (▴) and in IFN-α_2_b-treated cocultures with HMC (□) or with EMT-HMC (▵). Data are expressed as Europium counts × 10^6^ and are reported as the mean ± SEM obtained in three independent experiments set up in quadruplicates. IFN-α_2_b, interferon-α_2_b; HMC, human mesothelial cells; EMT, epithelial–mesenchymal transition; PEL, primary effusion lymphoma; TRAIL, tumor necrosis factor–related apoptosis-inducing ligand; PBGD, porphobilinogen deaminase.

We previously showed that murine mesothelial cells treated with mIFN-α_1_-induced apoptosis in cocultured PEL cells in a TRAIL-dependent manner 25, suggesting that the targeting of body cavity microenvironment may interfere with PEL growth. We therefore investigated whether the supportive activity exerted by mesothelium on PEL cells could be modulated by treatment with IFN-α_2_b. To this end, CRO-AP/3 cells were cultured with HMC or EMT-HMC pretreated with IFN-α_2_b for 6 h. Apoptosis was analyzed 24 h after coculture. As AZT was shown to sensitize PEL cells to TRAIL-mediated apoptosis 32, CRO-AP/3 cells were treated with AZT for 48 h before coculture. CRO-AP/3 cells cocultured with IFN-α_2_b-pretreated HMC or EMT-HMC showed a statistically significant increase in apoptotic cells compared with CRO-AP/3 cells cocultured with untreated HMC and EMT-HMC, respectively (Fig. 5A). A similar significant increase in apoptotic cells was obtained by directly adding IFN-α_2_b to the coculture to mimic the in vivo condition (Student *t* test, *P* ≤ 0.01, all treatment conditions vs. untreated control cocultures) (Fig. 5A). We next investigated whether the induced PEL cell death was also TRAIL-mediated in HMC undergoing EMT. The level of TRAIL expression, determined by quantitative RT-PCR, in IFN-α_2_b-treated EMT-HMC was found to be as high as that of treated HMC (Fig. 5B). Moreover, apoptosis induction in CRO-AP/3 cells, cocultured with IFN-α_2_b-stimulated HMC or EMT-HMC, was specifically inhibited by a blocking anti-TRAIL mAb (data not shown). These findings indicate that HMC and EMT-HMC activated by IFN-α_2_b trigger a TRAIL-mediated apoptosis in PEL cells.

A significant decrease in the number of BrdU-labeled proliferating CRO-AP/3 cells was observed in IFN-α_2_b-treated cocultures compared with untreated ones (ANOVA, *P *≤* *0.001, treated vs. untreated cocultures, all time points except 24 h), indicating that administration of this cytokine could also limit the proliferative advantage acquired by PEL cells in coculture with mesothelial cells (Fig. 5C).

These findings suggest that IFN-α_2_b treatment can interfere with the supportive activity exerted by mesothelium on lymphoma cells. Moreover, transdifferentiated mesothelial cells retain the ability to express high levels of TRAIL when stimulated with IFN-α_2_b, and to trigger a TRAIL-mediated programmed cell death in PEL cells.

## Discussion

Components of the local environment play important roles in cancer biology. Body cavity lymphomas grow in a site which in physiological conditions is a virtual cavity lined with a monolayer of mesothelial cells. These cells, along with immune-related components, are likely to engage in constant crosstalk with lymphomatous cells during tumor progression. The aim of our study was to examine the role of mesothelial cells in the progression of PEL, a lymphoma that preferentially arises in serous body cavities. We here demonstrate for the first time that PEL cells induce type 2 EMT in mesothelial cells. In vitro coculture systems showed that HMC transition to a myofibroblastic morphology was accompanied by downmodulation of cytokeratins and E-cadherin, responsible for the static and protective nature of an epithelial barrier, and upregulation of transcription repressors (Snail1, Slug, Zeb1, and Sip1) orchestrating the disassembling of cellular junctions. Moreover, IFA showed expression of α-SMA, one of the proteins that enhances cellular plasticity and contractility typical of mesenchymal cells (Fig. 1). Induction of EMT in HMC did not require cell-to-cell contact (Fig. 2) and was mainly triggered by TGF-β1, a powerful fibrogenic cytokine secreted at high levels by PEL cell lines (Fig. S3) 30,31. In fact, TGF-β1/IL-1β pretreatment followed by PEL cell coculture increased the expression of EMT-related markers in HMC with a clear additive effect (Fig. 1C), suggesting that the same mechanism/s might be at work in this condition. Furthermore, a highly selective inhibitor of a subset of activin receptor-like kinase receptors that include ALK5, the TGF-β type I receptor 26, abrogated Snail1 induction in a dose-dependent manner (Fig. 2). It is therefore conceivable that TGF-β1 signaling plays a major role in EMT induction in HMC by PEL cells, although other pathways may also be involved in the heterotypic interactions leading to EMT. It should be pointed out that other TGF-β1-producing cell lines (such as the HHV8-/EBV-BJAB cell line) induced EMT in HMC (data not shown), suggesting that this is not a PEL-specific mechanism, but a process shared by all TGF-β1-secreting lymphomatous cells growing in body cavities.

Type 2 EMT associated with chronic inflammation determines tissue fibrosis with architectural alteration. Indeed, patients undergoing peritoneal dialysis experience progressive damage of peritoneal membrane structure due to continuous EMT of HMC that eventually leads to an irreversible functional decline 11,14,18. In a similar manner, intracavitary growth of PEL cells continuously boosts EMT of HMC. We evidenced the occurrence of fibrosis in vivo using a mouse model recapitulating the aggressive course and liquid phase of peritoneal PEL 25. Histological analyses showed a progressive thickening of the serosal membranes in SCID/PEL mice, accompanied by inflammation and neoangiogenesis (Fig. 3). Increased blood vessel density was likely triggered by intracavitary accumulation of vascular endothelial growth factor, a mediator of neoangiogenesis and vascular permeability, secreted at high levels not only by PEL cells in vitro and in the peritoneal cavity of SCID/PEL mice 25 but also by myofibroblasts 33.

Serosal thickening was paralleled by loss of cytokeratin staining, and both are peculiar signs of fibrosis 33. This finding is in line with computed tomography scans and autopsy studies in PEL patients that revealed thickening and frequent lymphomatous infiltrations of serosal membranes, respectively 16,34. Extracavitary PEL onset during the course of intracavitary effusion likely represents the outcome of this process 35–37. It should be specified that a secondary tissue mass may develop in patients with a clinical course modulated by a therapeutic regimen, leading to effusion remission and relatively longer survival. Indeed, some cases of extracavitary PEL were demonstrated to be the relapse of the original tumor in a distinct anatomical site 22,35. Failure of serosal membrane function may thus explain the irreversible and rapid progression of PEL as well as the appearance of extracavitary masses in longer surviving patients. In line with this finding, our previous in vivo experiments showed that longer survival was associated with a significant reduction in ascites formation in IFN-α-treated mice, which, however, developed extracavitary masses 25.

HHV8 infection was recently found to induce EMT in lymphatic endothelial cells, thus explaining the origin of spindle cells, the characteristic neoplastic cells in lesions of Kaposi's sarcoma, another HHV8-associated tumor 38,39. Transdifferentiation program in lymphatic endothelial cells is mediated by the viral proteins vFLIP and vGPCR through the activation of Notch signaling 38. Preliminary experiments showed that short-term coculture with PEL cells led to a small fraction of latently infected mesothelial cells (2–5%, data not shown). Therefore, although HHV8 infection might potentially mediate EMT in infected HMC, our preliminary findings suggest that viral infection per se takes part marginally, if at all, in EMT induction in mesothelial cells.

Although PEL cells have full growth autonomy, the coculture significantly increased the resistance to apoptosis and contributed a proliferative advantage to PEL cells (Fig. 4). Uninfected B and T neoplastic cell lines displayed a similar increase in proliferation during HMC coculture (data not shown), indicating that growth support may be extended to other neoplastic effusions growing in body cavities. Furthermore, it should be mentioned that, in the occasional setting of primary extracavitary masses, other components of the tumor microenvironment may not only sustain proliferation and survival of PEL cells but could also restore their preferential tropism for body cavities and growth in liquid phase. Of note, HMC transdifferentiation did not alter the supportive activity. As myofibroblastic conversion reprograms the gene expression profile 40, modulation of PEL cell turnover in EMT-HMC could involve different signals and intracellular pathways. Ongoing experiments are aimed at dissecting the driving mechanisms of this process, as the identification of the involved molecular pathways may furnish new attractive targets that could be exploited therapeutically.

The combination of AZT and IFN-α was previously shown to induce apoptosis in PEL-derived and other herpesvirus-associated lymphoma cell lines, and was successfully exploited to treat a patient with AIDS-associated PEL 41,42. This combined treatment was also shown to be effective in a distinct type of virus-induced lymphoma, leading to increased survival in patients with the leukemic subtypes of human T-cell lymphotropic virus type 1–associated adult T-cell leukemia/lymphoma 43. We previously demonstrated that mesothelial cells could contribute to the antineoplastic activity of intracavitary IFN-α treatment by exposing TRAIL and inducing programmed cell death in PEL cells 25. We extended this finding to mesothelial cells undergoing EMT (Fig. 5 and data not shown) that were found to express high levels of TRAIL after IFN-α_2_b treatment and to retain a TRAIL-mediated tumoricidal activity. Moreover, direct IFN-α_2_b treatment of the coculture was also found to decrease the proliferative advantage of PEL cells. Collectively, these data indicate that IFN-α_2_b treatment may counteract the supportive activity exerted by the mesothelium and that transdifferentiated mesothelial cells retain the ability to respond to this cytokine.

In conclusion, this study demonstrates that intracavitary growth of PEL induces EMT in the mesothelium, and that this pathogenic mechanism may play a role in PEL progression. We also show that mesothelial cells and their transdifferentiated cognates generate an environment that confers a proliferative advantage and increased survival on neoplastic cells, indicating that any intracavitary treatment directed at PEL cells has to overcome a highly protective environment. Our findings suggest that a therapy targeted to the microenvironment, which specifically neutralizes the prosurvival stimuli furnished by mesothelial cells, may provide a valuable strategy to control PEL progression and, possibly, other neoplastic effusions growing in body cavities. Moreover, drugs that counteract EMT may ameliorate treatment of PEL, possibly by preventing relapse.

## Supporting Information

**Data S1.** Supplemental Materials and Methods **Figure S1.** Immunophenotypic characterization of human mesothelial cells (HMC). (A) Early-passage primary HMC were analyzed for the expression of intracellular and cell surface markers by flow cytometric analyses. Data are expressed as percentage of positive cells for the indicated antigen and are reported as mean ± SD measured in five different primary cultures. HMC express at high levels pan cytokeratin, E-cadherin, CD44, and CD54, at moderate levels mesothelin, and lack expression of α-SMA, CD14, and Fsp-1. (B) Representative flow cytometry plots obtained from a first-passage culture of HMC. Dotted lines are cells labeled with isotype-matched antibodies. **Figure S2.** Coculture with BCBL-1 and CRO-AP/2 cells induced EMT in mesothelial cells. (A) Phase-contrast images of representative HMC after coculture with BCBL-1 and CRO-AP/2 cells. HMC acquired a myofibroblastic phenotype, with elongated shape and multilayered growth after coculture with CRO-AP/2 and BCBL-1 cells. (B) Relative expression of pan cytokeratin (pCK) and Snail1 transcripts in HMC after coculture with PEL cell lines. Quantitative RT-PCR analyses were performed on RNA extracted from HMC cultured for 6 days in standard conditions, and from HMC cocultured for 6 days with BCBL-1 or CRO-AP/2 cells. Human PBGD was used as housekeeping gene. Expression levels of pCK and Snail1 transcripts are reported relative to expression levels measured in HMC. Data are reported as mean ± SD. Downregulation of pCK and upregulation of Snail1 were measured in HMC cocultured with CRO-AP/2 and BCBL-1 cells, suggesting that all PEL cell lines may induce EMT in cocultured HMC. **Figure S3.** Analysis of expression and release of TGF-α1 in PEL-derived cell lines. (A) Relative expression of TGFα1 was analyzed by quantitative RT-PCR in RNA extracted from CRO-AP/3, CRO-AP/2, and BCBL-1 cell lines. PBGD was used as housekeeping gene and expression levels are reported relative to levels measured in 239 H cells. Data are expressed as mean ± SD. (B) Measurement of TGF-α1 levels in culture supernatants of PEL cell lines. Data are expressed as picograms per milliliter, and each histogram represents the mean ± SEM of data obtained in two independent measurements set up in duplicate. PEL cells express and release high levels of TGF-α1, suggesting that EMT in HMC may be induced by this factor. **Table S1.** Designation and sequence of primers used in qualitative and quantitative RT-PCR.
