# Supplementary material for: Crosstalk between the mesothelium and lymphomatous cells: insight into the mechanisms involved in the progression of body cavity lymphomas
Source: Cancer Med. 2013 Nov 19;3(1):1–13. doi: 10.1002/cam4.159 (PMC3930384; doi:10.1002/cam4.159)
Supplement: Supplementary file 1 — Data S1. Supplemental Materials and Methods Figure S1. Immunophenotypic characterization of human mesothelial cells (HMC). (A) Early-passage primary HMC were analyzed for the expression of intracellular and cell surface markers by flow cytometric analyses. Data are expressed as percentage of positive cells for the indicated antigen and are reported as mean ± SD measured in five different primary cultures. HMC express at high levels pan cytokeratin, E-cadherin, CD44, and CD54, at moderate levels mesothelin, and lack expression of α-SMA, CD14, and Fsp-1. (B) Representative flow cytometry plots obtained from a first-passage culture of HMC. Dotted lines are cells labeled with isotype-matched antibodies. Figure S2. Coculture with BCBL-1 and CRO-AP/2 cells induced EMT in mesothelial cells. (A) Phase-contrast images of representative HMC after coculture with BCBL-1 and CRO-AP/2 cells. HMC acquired a myofibroblastic phenotype, with elongated shape and multilayered growth after coculture with CRO-AP/2 and BCBL-1 cells. (B) Relative expression of pan cytokeratin (pCK) and Snail1 transcripts in HMC after coculture with PEL cell lines. Quantitative RT-PCR analyses were performed on RNA extracted from HMC cultured for 6 days in standard conditions, and from HMC cocultured for 6 days with BCBL-1 or CRO-AP/2 cells. Human PBGD was used as housekeeping gene. Expression levels of pCK and Snail1 transcripts are reported relative to expression levels measured in HMC. Data are reported as mean ± SD. Downregulation of pCK and upregulation of Snail1 were measured in HMC cocultured with CRO-AP/2 and BCBL-1 cells, suggesting that all PEL cell lines may induce EMT in cocultured HMC. Figure S3. Analysis of expression and release of TGF-α1 in PEL-derived cell lines. (A) Relative expression of TGFα1 was analyzed by quantitative RT-PCR in RNA extracted from CRO-AP/3, CRO-AP/2, and BCBL-1 cell lines. PBGD was used as housekeeping gene and expression levels are reported relative to levels measured [file cam40003-0001-sd1.pdf]

Supporting Information

Supplemental Materials and Methods

Quantitative assays for TGF-β1

To quantify TGF-β1 expressed and released in culture supernatants by PEL-derived cell lines, cells were suspended at 10^6/mL in complete medium containing 2% FCS, and cells and supernatants were collected after 24 hours. Quantitative RT-PCR for TGF-β1 expression is described in "Molecular analyses" section. The amount of TGF-β1 in culture supernatants was assessed by an enzyme-linked immunosorbent assay (Human TGF-β1 Instant ELISA, eBioscience, San Diego, CA, USA). The level of TGF-β1 in control medium was subtracted from that of each sample to account for TGF-β1 present in FCS. Two independent measurements set up in duplicates were done, and data were shown as mean ± SEM.

Antibodies, flow cytometry and IFA

To detect pan cytokeratin (pCK) and α-smooth muscle actin (α-SMA), human mesothelial cells (HMC) were fixed, rendered permeable and incubated with a mouse anti-pCK monoclonal antibody (mAb) mixture (mouse ascites fluid, Sigma-Aldrich) and a rabbit polyclonal antibody to α-SMA (Abcam, Cambridge, UK), and then with an Alexa 594-conjugated goat anti-mouse immunoglobulin G (IgG; heavy and light chains [H+L]) and an Alexa 488-conjugated chicken anti-rabbit IgG (H+L) (Molecular Probes, Eugene, OR, USA). HMC were also stained with a goat polyclonal Ab to E-cadherin (R&D Systems), and then with an Alexa 594-conjugated rabbit anti-goat IgG (H+L) (Molecular Probes). To evaluate the expression of mesothelin and fibroblast surface protein 1 (Fsp-1), a mouse mAb to mesothelin or to Fsp-1 (GeneTex, San Antonio, TX, USA) was used as primary Ab, and an Alexa 488-conjugated goat anti-mouse IgG (H+L) or IgM (Molecular Probes), respectively, was used as secondary Ab. HMC staining was also performed using a phycoerythrin (PE)-conjugated mouse mAb anti-CD14 (AbD Serotec, Oxford, UK), a PE-conjugated mouse mAb to CD54/ICAM-1 (eBioscience, San Diego, CA, USA), and a PE-conjugated rat mAb to CD44 (BioLegend, San Diego, CA, USA). All antibodies were used diluted according to the manufacturers' instructions in PBS-3% BSA. Isotype-matched irrelevant mAbs or secondary mAbs were always used in parallel as negative controls. Analyses were done using a LSR-II flow cytometer (BD Biosciences, Franklin Lakes, NJ, USA) equipped with a 488-, 405-, 640-, and 561-nm laser and an EPICS XL flow cytometer (Coulter Electronics, Hialeah, FL, USA).

To assess the expression of pCK and α-SMA by IFA, HMC were seeded on coverslips previously coated with poly-L-lysine and cocultured with PEL cells or treated with TGF-β1/IL-1β or exposed to the combined condition. Cells were fixed, rendered permeable and incubated with primary antibodies diluted in PBS-2% FCS for 40 minutes at 37°C, washed three times with PBS, and incubated with appropriate secondary antibodies, followed by 4',6-diamino-2-phenylindole (DAPI, Sigma-Aldrich) counterstaining. Stained cells were visualized using a fluorescence microscope (Vico, Nikon, Melville, NY, USA) with a 20x/0.50 NA objective and images were acquired as stated above.

Immunohistochemical analyses.

Ki67 staining was performed in immunohistochemistry using a rabbit anti-mouse Ki67 antibody (Novocastra Laboratories, Newcastle, UK). Immunostaining was carried out using the avidin-biotin-peroxidase complex technique (Vectastain ABC kit; Vector Labs, Burlingame, CA, USA), and 3,3'-diaminobenzidine (DAB kit, Dako, Glostrup, Denmark) was used as a chromogen substrate. Counterstain was performed with haematoxylin and images were acquired with a digital colour camera (DFC295, Leica Microsystems Ltd, Wetzlar, Germany) using an optical microscope (Olympus BX40, Melville, NY, USA).

Apoptosis analysis and proliferation assay

Spontaneous and induced apoptosis was evaluated at different time points in CRO-AP/3, BCBL-1 and CRO-AP/2 cells suspended at 0.5-1 x 10^5/mL and cocultured with or without HMC, or HMC undergoing EMT (EMT-HMC). EMT-HMC were obtained by TGF-β1/IL-1β treatment for 96 hours. Apoptosis was induced in PEL cell lines by serum deprivation (complete medium with 5-10% FCS). To assess the proapoptotic activity of IFN-α_2b in coculture, HMC or EMT-HMC were treated with 6000 international units (IU)/mL recombinant IFN-α_2b (IntronA, Schering Plough, Kenilworth, NJ, USA) for 6 hours, then washed and cocultured with CRO-AP/3 cells for 24 hours. CRO-AP/3 cells were sensitized to TRAIL-mediated apoptosis by treatment with 10 μg/mL azidothymidine (AZT, Sigma-Aldrich, Munich, Germany) for 48 hours before coculture. To mimic *in vivo* conditions, IFN-α_2b was added at the beginning of the coculture and apoptosis was measured after 24 hours. Apoptosis was analyzed by flow cytometry after staining with annexin V (Annexin-V-FLUOS staining kit, Roche Diagnostic, Indianapolis, IN, USA). To block TRAIL-induced apoptosis, cocultures were performed by adding an anti–TRAIL mAb or an isotype control

(5 µg/mL) [clone RIK-2, low-endotoxin azide-free (LEAF)–purified anti–human TRAIL, or LEAF-purified isotype control; BioLegend, San Diego, CA, USA] to IFN-α_2b-treated HMC or EMT-HMC before the coculture. The proliferative capability of PEL cell lines was analyzed by 5-bromo-2'-deoxyuridine (BrdU) incorporation measured at different time points using an Europium-labeled anti-BrdU antibody (Delfia cell proliferation assay, PerkinElmer, Cambridge, UK) by time-resolved fluorescence using a VICTOR™ X4 Multilabel Plate Reader (PerkinElmer) at 615 nm. Proliferation was measured in PEL-derived cell lines (2-4 x 10^4/mL) plated in empty wells and on HMC or EMT-HMC seeded in 96-well flat-bottomed plates (ViewPlate, PerkinElmer). Proliferation was also measured in HMC and EMT-HMC alone. Each culture condition was set up in triplicate or quadruplicate and incubated with BrdU at each time point. To analyze the cytostatic activity of IFN-α_2b, experiments were set up as described before, and IFN-α_2b (6000 IU/mL) was added to the coculture. Results were reported as the mean of Europium counts ± SEM of three independent experiments.

Supplemental Table S1

Table S1: Designation and sequence of primers used in qualitative and quantitative RT-PCR.

**Primer**	**Sequence (5’→3’)**
Snail1B/For	TCAAAGGCCATGTCCGGACCCA[1]
Snail1B/Rev	GGAGCTTCCCAGTGAGTCTG[1]
PBGD/For	TGCCCTGGAGAAGAATGAAG
PBGD/Rev	CAGCATCATGAGGGTTTTCC
pCK/For	AACIGIATGATCCAGAGGC
pCK/Rev	CATCCTTGAGGGCCAICTC
E-cad2/For	GCCGAGAGCTACACGTTCAC[2]
E-cad2/Rev	GTCGAGGGAAAAATAGGCTG[2]
Snail1/For	CTGCAGGACTCTAATCCAGA
Snail1/Rev	ATCTCCGGAGGTGGGATG
Zeb1/For	AGACATGTGACGCAGTCTGG
Zeb1/Rev	TTGCAGTTTGGGCATTCATA
Zeb2/For	CAAGAGGCGCAAACAAGC
Zeb2/Rev	GGTTGGCAATACCGTCATCC
Slug/For	TGGTTGCTTCAAGGACACAT
Slug/Rev	GTTGCAGTGAGGGCAAGAA
α-SMA/For	GCTGTTTTCCCATCCATTGT
α-SMA/Rev	TGGTGATGATGCCATGTTCT
TGF-β1/For	CCCTGGACACCAACTATTGC
TGF-β1/Rev	AAGTTGGCATGGTAGCCCTT
hTRAIL/For	TTCACAGTGCTCCTGCAGTC
hTRAIL/Rev	AGCAATGCCACTTTTGGAGT

[1] Primers used in qualitative RT-PCR.
[2] Primers used in qualitative and quantitative RT-PCR.

Supplemental Figures

Figure S1

a

Marker	Expression on HMC (%)
pan cytokeratin	95 ± 5
E-cadherin	87 ± 2
CD44	70 ± 5
CD54	85 ± 10
Mesothelin	20 ± 5
α-SMA	1 ± 0.5
CD14	0 ± 0.1
Fsp-1	0 ± 0.1

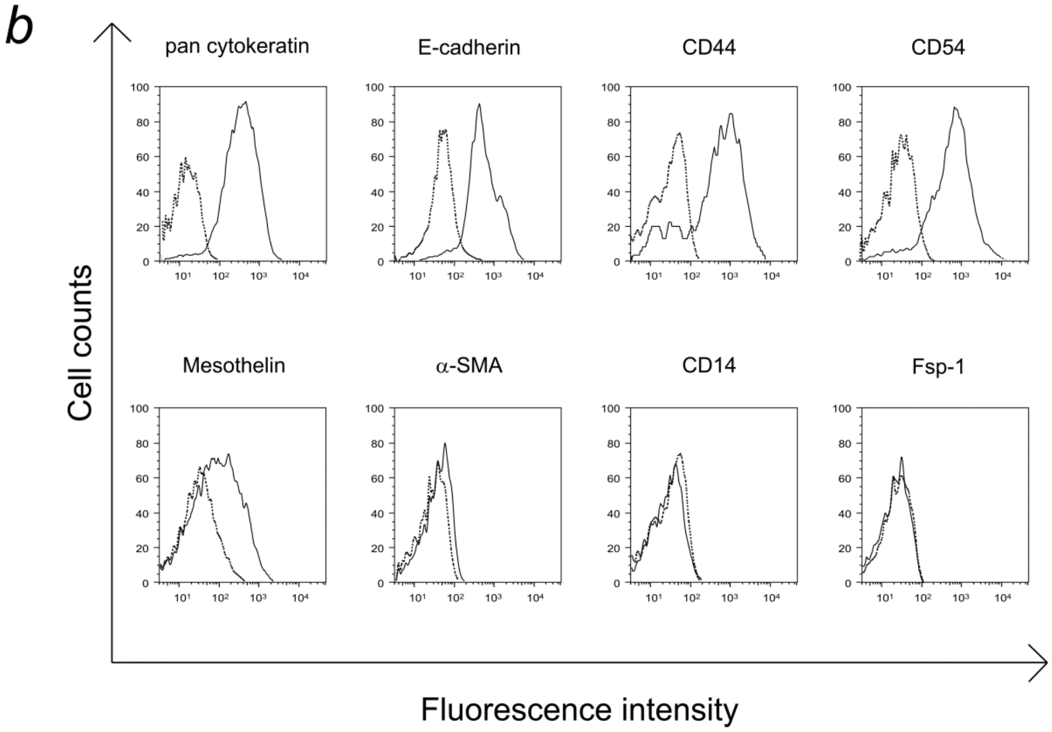

Figure S1: Immunophenotypic characterization of human mesothelial cells (HMC). (*a*) Early-passage primary HMC were analyzed for the expression of intracellular and cell surface markers by flow cytometric analyses. Data are expressed as percentage of positive cells for the indicated antigen and are reported as mean ± SD measured in 5 different primary cultures. HMC express at high levels pan cytokeratin, E-cadherin, CD44 and CD54, at moderate levels mesothelin, and lack expression of α-SMA, CD14 and Fsp-1. (*b*) Representative flow cytometry plots obtained from a first-passage culture of HMC. Dotted lines are cells labelled with isotype-matched antibodies.

Figure S2

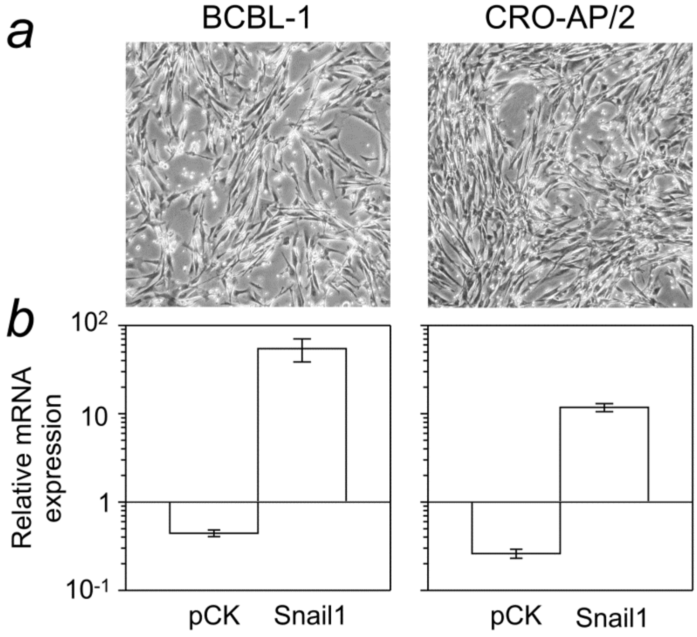

Figure S2: Coculture with BCBL-1 and CRO-AP/2 cells induced EMT in mesothelial cells. (*a*) Phase-contrast images of representative HMC after coculture with BCBL-1 and CRO-AP/2 cells. HMC acquired a myofibroblastic phenotype, with elongated shape and multi-layered growth after coculture with CRO-AP/2 and BCBL-1 cells. (*b*) Relative expression of pan cytokeratin (pCK) and Snail1 transcripts in HMC after coculture with PEL cell lines. Quantitative RT-PCR analyses were performed on RNA extracted from HMC cultured for 6 days in standard conditions, and from HMC cocultured for 6 days with BCBL-1 or CRO-AP/2 cells. Human PBGD was used as housekeeping gene. Expression levels of pCK and Snail1 transcripts are reported relative to expression levels measured in HMC. Data are reported as mean ± SD. Downregulation of pCK and upregulation of Snail1 was measured in HMC cocultured with CRO-AP/2 and BCBL-1 cells, suggesting that all PEL cell lines may induce EMT in cocultured HMC.

Figure S3

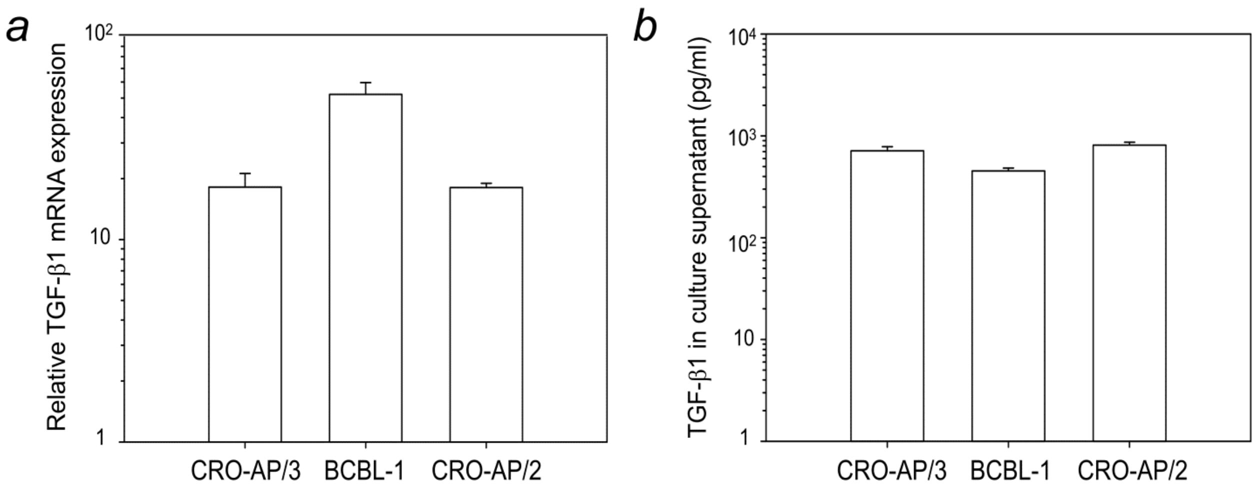

Figure S3: Analysis of expression and release of TGF-β1 in PEL-derived cell lines. (*a*) Relative expression of TGF-β1 was analyzed by quantitative RT-PCR in RNA extracted from CRO-AP/3, CRO-AP/2 and BCBL-1 cell lines. PBGD was used as housekeeping gene and expression levels are reported relative to levels measured in 239 H cells. Data are expressed as mean ± SD. (*b*) Measurement of TGF-β1 levels in culture supernatants of PEL cell lines. Data are expressed as picograms per milliliter, and each histogram represents the mean ± SEM of data obtained in two independent measurements set up in duplicate. PEL cells express and release high levels of TGF-β1, suggesting that EMT in HMC may be induced by this factor.
